# A prospective study of decline in fat free mass and skeletal muscle strength in chronic obstructive pulmonary disease

**DOI:** 10.1186/1465-9921-8-25

**Published:** 2007-03-13

**Authors:** Nicholas S Hopkinson, Rachel C Tennant, Mark J Dayer, Elisabeth B Swallow, Trevor T Hansel, John Moxham, Michael I Polkey

**Affiliations:** 1 Respiratory Muscle Laboratory, Royal Brompton Hospital, Fulham Rd, London SW3 6NP, UK; 2 Clinical Studies Unit National Heart and Lung Institute, Royal Brompton Hospital, Fulham Rd, London SW3 6NP, UK; 3 Respiratory Muscle Laboratory, Guy's King's and St Thomas' School of Medicine, King's College Hospital, Denmark Hill, London SE5 9RS, UK

## Abstract

**Background:**

Skeletal muscle depletion is an important complication of chronic obstructive pulmonary disease (COPD) but little prospective data exists about the rate at which it occurs and the factors that promote its development. We therefore prospectively investigated the impact of disease severity, exacerbation frequency and treatment with corticosteroids on change in body composition and maximum isometric quadriceps strength (QMVC) over one year.

**Methods:**

64 patients with stable COPD (FEV_1 _mean (SD) 35.8(18.4) %predicted) were recruited from clinic and studied on two occasions one year apart. Fat free mass was determined using bioelectrical impedance analysis and a disease specific regression equation.

**Results:**

QMVC fell from 34.8(1.5) kg to 33.3(1.5) kg (p = 0.04). The decline in quadriceps strength was greatest in those with the highest strength at baseline (R -0.28 p = 0.02) and was not correlated with lung function, exacerbation frequency or steroid treatment. Decline in fat free mass was similarly higher in those with largest FFM at baseline (R = -0.31 p = 0.01) but was more strongly correlated with greater gas trapping (R = -0.4 p = 0.001). Patients with frequent exacerbations (>1 per year) (n = 36) experienced a greater decline in fat free mass compared to infrequent exacerbators (n = 28) -1.3(3.7)kg vs. +1.2(3.1)kg (p = 0.005), as did patients on maintenance oral steroids (n = 8) -2.8(3.3) kg vs. +0.2(3.5) kg (p = 0.024) whereas in those who stopped smoking (n = 7) fat free mass increased; +2.7(3.1) kg vs. -0.51(3.5) kg (p = 0.026).

**Conclusion:**

Decline in fat free mass in COPD is associated with worse lung function, continued cigarette consumption and frequent exacerbations. Factors predicting progression of quadriceps weakness could not be identified from the present cohort.

## Background

It is well established that chronic obstructive pulmonary disease (COPD) has systemic consequences, one of which is fat free mass depletion [1], which is independently associated with excess mortality [2] and impaired quality of life [3]. A number of studies have identified weight loss and low BMI as independent predictors of mortality [4,5] and patients who failed to gain weight after a program of nutritional support also had a worse prognosis [6]. Skeletal muscle is a major component of fat free mass and skeletal muscle depletion is itself associated with reduced exercise capacity [7-9], while thigh muscle bulk has been found to predict survival [10]. Very recently quadriceps strength itself has been demonstrated to be a predictor of mortality in COPD [11]. A variety of mechanisms have been postulated, including disuse atrophy, poor nutrition, oral corticosteroid treatment [12], systemic inflammatory mediators [1] and more recently, genetic factors [13,14].

However no prospective data are available regarding the rate of decline of strength in COPD and the factors that are responsible for it. Short term studies have shown that exacerbations of COPD are associated with increased inflammatory mediators and acute and partially reversible reductions in both quadriceps [15] and handgrip strength [16]. Since exacerbations are also associated with immobility, negative nitrogen balance [16] and reduced mobility, it seems reasonable to hypothesize that the development of skeletal muscle depletion over time would be associated with exacerbation frequency.

Based on this interpretation of the literature, this study evaluated various *a priori *determined factors to determine their utility in predicting decline in fat free mass and quadriceps strength. The factors to be evaluated were indices of lung function, oral corticosteroid exposure and having frequent exacerbations

## Methods

Patients were recruited from clinics at The Royal Brompton Hospital if they had COPD, defined according to the GOLD guidelines [17], without significant diagnosed co-morbidity (including heart failure, neuromuscular disease and other conditions likely to impact on skeletal muscle) or evidence of exacerbation in the preceding month. The Royal Brompton and Harefield Hospitals' Research Ethics Committee approved the study, which was conducted in accordance with the Helsinki declaration and all patients gave their written informed consent. Clinical information including treatment, health related quality of life (St George' Respiratory Questionnaire [18]), number of exacerbations in the previous year (defined as discrete episodes of worsening of respiratory symptoms leading to treatment with antibiotics), and average daily dose (ADD) of oral prednisone received was obtained from patients through a structured interview with reference to their hospital records relating to any outpatient appointments in the intervening period. Patients were studied at baseline and tests repeated at a one year follow up visit. None of the patients took part in a pulmonary rehabilitation course during the follow up period. Baseline data from some of the subjects in this study has been published previously [11,13,14,19].

Spirometry was obtained using a pneumotachograph with flow integration, lung volumes by whole body plethysmography and gas transfer with a single breath technique (CompactLab System, Jaeger, Germany). Blood gas tensions were measured in arterialised earlobe capillary samples. Fat free mass (FFM) was determined using bioelectrical impedance analysis (Bodystat 1500, Bodystat, Isle Of Man, UK) and a disease specific regression equation [20].

Maximum isometric quadriceps strength (QMVC) was measured with subjects seated, trying to extend their dominant leg as hard as possible against an inextensible strap connecting their ankle to a strain gauge (Strainstall Ltd, Cowes, UK) [21]. The signal was amplified and passed to a computer running LabView 4 software (National Instruments, Austin, Texas). The force generated was visible to subject and investigator for positive feedback and repeated efforts were made with vigorous encouragement until there was no improvement in performance. Efforts were sustained for at least 5 seconds. Subjects rested for about 30 seconds between each contraction. According to convention, values were normalised for body weight (QMVC %predicted) [21].

To assess respiratory muscle strength, maximum sniff nasal (SNiP) and static expiratory (PEmax) mouth pressures were also determined [22,23].

### Statistical analysis

The main outcome measures were change in FFM and QMVC. Analysis was performed using StatView 5.0 (Abacus concepts, Inc., Berkeley, CA, USA) and focused on the effect of exacerbation rate, oral steroid exposure and disease severity (%predicted values of gas transfer (TL_CO_), forced expiratory volume in one second (FEV_1_), and functional residual capacity (FRC). Frequent exacerbators were defined *a priori *as those having two or more exacerbations per year consistent with international guidelines [24]. Change in FFM and QMVC were related both to baseline patient characteristics and to change in these parameters over time using forward stepwise regression analysis including parameters with a p value of < 0.1 by univariate analysis. A p value of < 0.05 was taken to be significant.

## Results

### Follow up

Of 109 patients studied at baseline, five (4.6%) were actively excluded from analysis because they developed significant co morbidity during the year (malignancy or cardiac disease) and nine (8.3%) patients died during the follow up period. We did not seek to follow up two patients who had moved to a distant part of the country and were no longer patients of the hospital. Thirteen (11.9%) declined to come back for further testing. Of these, two felt too unwell to come, two declined to travel and nine gave no specific reason. For 16 of the remaining 80 patients, clinical data, lung function and weight were available but not measures of strength or fat free mass. This was primarily for logistical reasons, for example difficulty coordinating visits to the lab with patients' clinic appointments. The group that was not followed up did not differ significantly from those who were, in terms of baseline lung function, GOLD stage, strength, body composition, or ADD steroids and exacerbation rate in the year prior to the start of the study (Table 1).

**Table 1:** Strength and body composition at baseline and one year follow up

	Subjects not followed up n = 43	Baseline n = 64	One Year follow up n = 64
QMVC (kg)	32.2 (12.8)	34.8 (1.5)	33.3 (1.5)*
QMVC %predicted	64.6 (20.8)	66.3 (17.9)	62.3 (17.7)*
BMI (kgm^-2^)	23.5 (4.4)	24.3 (5.2)	24.7 (5.4)
Weight (kg)	67.1 (15.0)	70.5 (15.7)	71.4 (15.9)
FFM (kg)	46.7 (8.3)	47.5 (8.3)	47.3 (7.9)
SNiP (cmH_2_O)	61.5 (19.6)	67.4 (19.5)	69.9 (21.9)
PEmax (cmH_2_O)	89.2 (30.2)	98.9 (43.9)	100.0 (44.3)
Age (years)	65.5 (9.9)	62.0 (9.4)	
FEV_1 _%predicted	40.5 (17.9)	36.0 (18.4)	36.3 (19.4)
TL_CO _%predicted	39.8 (19.9)	40.1 (19.3)	40.5 (20.0)
FRC %predicted	181 (32)	176 (41)	175 (39)
PaCO_2 _(kPa)	5.2 (1.1)	5.2 (0.9)	5.2 (0.9)
PaO_2 _(kPa)	9.4 (1.4)	9.4 (1.6)	9.2 (1.4)

QMVC – quadriceps maximum voluntary contraction, FFM – fat free mass, SNiP – sniff nasal pressure, PEmax – maximum expiratory pressure, FEV_1 _– forced expiratory volume in one second, TL_CO _– carbon monoxide transfer factor, FRC functional residual capacity. Mean (SD). There was no significant difference at baseline between patients who were or were not followed up at one year. *p < 0.05 vs baseline.

The results given henceforth in this paper are for the 64 patients in whom both baseline and follow up measurements of FFM were performed. 22 (34 %) were female, mean cigarette exposure was 47 (28) pack years and 16 (25%) were continued smokers. At baseline, 43 (67%) were using inhaled steroids, seven (10%) were taking regular oral steroids (≤ 10 mg prednisone per day), 19 (30%) had a nebuliser and 12 (19%) were on long term home oxygen therapy. 1,13,15,35 patients were in GOLD stages 1 to 4 respectively. Other baseline characteristics are given in Table 1.

### Factors associated with strength and body composition at baseline

At baseline 23 (36%) of the patients studied had fat free mass depletion (defined as a fat free mass index (FFMI) < 15 kg.m^-2 ^for women or < 16 kg.m^-2 ^for men). The FFM deplete group also had significantly worse gas transfer; %predicted TL_CO _30.9 (16.8) vs. 44.5 (19.4) (p = 0.008), but did not differ significantly in other lung function parameters or in terms of oral steroid exposure or reported exacerbation rate in the year prior to the start of the study.

Patients with FFM depletion had significantly weaker quadriceps QMVC 27.5 (9.3) kg vs. 38.7 (11.3) kg (p = 0.0002) and expiratory muscle strength MEP 79.1(31.2) cmH_2_O vs. 109.7 (46.4) cmH_2_O (p = 0.016) but SNiP did not differ significantly 64.6 (20.0) cmH_2_O vs. 68.3 (19.5) cmH_2_O (p = 0.5).

Quadriceps strength at baseline was significantly correlated with FFM (r^2 ^0.35 p < 0.0001) and %predicted TL_CO _(r^2 ^0.1 p = 0.04), but not with lung volume or airflow obstruction, nor with having frequent exacerbations, ADD prednisone in the preceding year and smoking history. Only FFM was retained as an independent variable in stepwise regression analysis.

### Change in quadriceps strength during follow up

Over the course of a year mean (SD) QMVC fell significantly from 34.8 (1.5) kg 66.3 (17.9) % predicted, to 33.3 (1.5) kg 62.3 (17.7) %predicted (p = 0.04 and 0.009 respectively) (Table 1).

Decline in QMVC was only correlated with baseline QMVC (r^2 ^0.1 p = 0.025) with the greatest decline in the patients who were strongest at baseline. 36 (56%) of the patients were defined as frequent exacerbators. Decline in QMVC in this cohort was not associated with disease severity, having frequent exacerbations or corticosteroid treatment.

### Change in fat free mass during follow up

**Figure 1:**
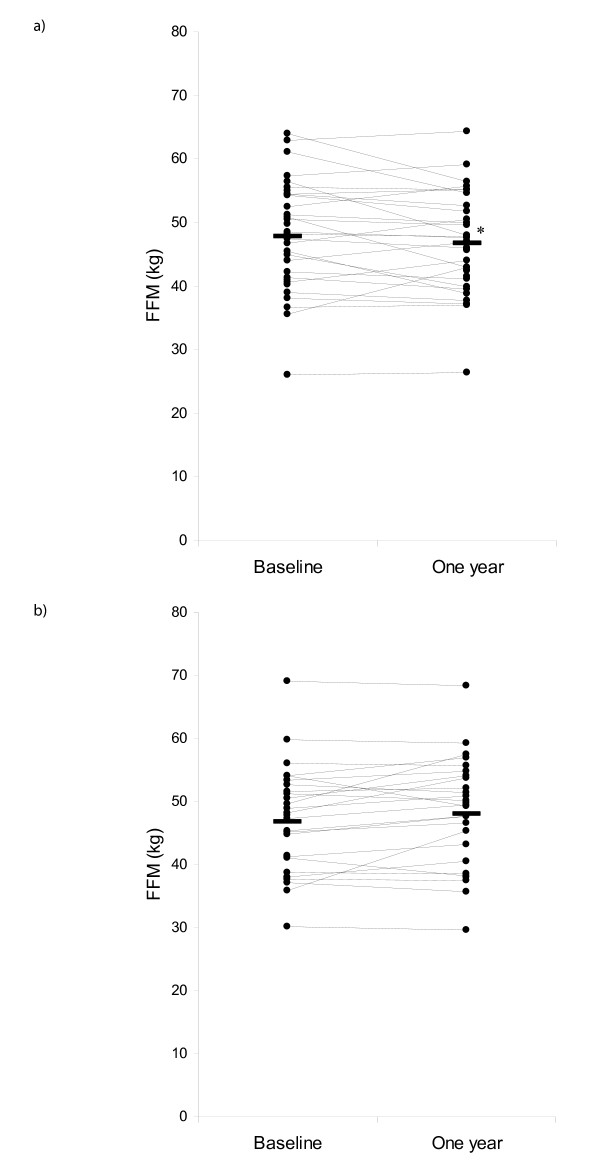
**Change in fat free mass over one year in frequent (≥ 2/yr)(a) and infrequent (b) exacerbators**. Horizontal bars represent mean values. * p = 0.005 comparing change in FFM in frequent and infrequent exacerbators.

Decline in FFM was associated with a higher baseline fat free mass, worse quality of life (judged as higher SGRQ total score), worse lung function, being on maintenance prednisone and having frequent exacerbations (Table 2) (Figure 1).

**Table 2:** Factors correlated with change in fat free mass

	R	p
Baseline FFM	-0.3	0.013*
SGRQ total score	-0.27	0.04*
FEV_1 _(%predicted)	0.27	0.03*
TL_CO _(%predicted)	0.19	0.15
FRC (%predicted)	-0.42	0.008*
ADD Prednisone	-0.24	0.06
Frequent exacerbations	-0.34	0.006*

All values given are those measured at baseline except the exacerbation rate which refers to exacerbations occurring during the period of follow up. FFM – fat free mass, SGRQ – St George's respiratory questionnaire, FEV_1 _– forced expiratory volume in one second, TL_CO _– carbon monoxide transfer factor, FRC – functional residual capacity. R values are for univariate analysis. Mean (SD). *p < 0.05.

The 8 patients on long term maintenance prednisone at the one year follow up visit had a significantly greater decline in FFM compared to the rest of the group; -2.8(3.3) kg vs. +0.2(3.5) kg (p = 0.024). The ADD prednisone received during follow up was median (range) 0 (0–26.1) mg.day^-1^. Changes in FFM did not differ significantly between those who had (n = 32) or had not received any prednisone during the year, being -0.86 (3.2) kg vs. +0.54 (3.8) kg (p = 0.12) respectively. In addition, to look for evidence of a dose response effect, ADD prednisone was log transformed to normalize it (this also excludes zero values). There was no correlation between log transformed ADD prednisone and change in FFM. Patients on maintenance oral prednisone were more likely to be frequent exacerbators (Chi^2 ^12.4 p < 0.001).

Seven patients stopped smoking during the follow up period and experienced a significant increase in FFM compared to the rest of the patients studied; +2.7 (3.1)kg vs. -0.51(3.5)kg (p = 0.026). Comparing quitters to continued smokers (n = 9) the change in FFM was +2.7(3.1)kg vs +0.6(1.0)kg (p = 0.08).

By stepwise regression analysis, percent predicted FRC, being on maintenance prednisone, smoking cessation and baseline FFM were retained, with the equation ΔFFM = -0.165 × (FFM) – 0.037 × (%predicted FRC) + 6.5 × (smoking cessation = 1) - 3.3 × (maintenance prednisone = 1) explaining 46% of the variance (p < 0.0001).

### Respiratory muscle strength

Measures of respiratory muscle strength are given in Table 1. There was no relationship between changes in SNiP or PEmax and baseline respiratory muscle strength, pulmonary function, or steroid exposure and exacerbation frequency analyzed in the same manner as for quadriceps strength.

## Discussion

This study investigated changes in fat free mass and skeletal muscle strength in a cohort of patients with COPD over one year's follow up. Skeletal muscle depletion was common at baseline and was associated with a more severe impairment in gas transfer. During follow up, decline in fat free mass was independently associated with more marked gas trapping, a higher FFM at baseline and use of maintenance oral corticosteroids, whereas FFM increased in patients who stopped smoking. An association between frequent exacerbations and decline in FFM was not retained as an independent correlate.

At baseline, quadriceps weakness was most marked in those with reduced fat free mass and declined further over the course of a year. The only parameter predicting decline in QMVC during follow up was QMVC at baseline. Of note the mean decline in QMVC was 4.3% which is significantly more than the 1–2% per annum anticipated in a healthy aging population [25-27]. This greater decrease is of considerable interest given that an association has been demonstrated between quadriceps strength and mortality in studies of healthy elderly subjects [28,29]. In the former study a reduction in quadriceps force of 38 NM (about twice that observed in our study) was associated with a hazard ratio for death of 1.51 in men and 1.65 in women. Recently an association between quadriceps strength and mortality has also been found in patients with COPD which was independent of lung function [11].

### Methodological issues

Follow up data was not available for all of the patients studied at baseline which could be a source of bias. Even in shorter term studies of muscle strength in COPD follow up has been problematic [15]. A number of arguments can be made to offset the significance of this however. Since the purpose of the study was to examine the natural history of decline in patients with COPD, we excluded those who developed significant co morbidity such as cancer or cardiovascular disease which would themselves have influenced strength or fat free mass. Moreover in a significant proportion of those not followed up the reasons were logistical, to do with coordinating laboratory visits with clinic appointments and therefore 'random' and unlikely to be a source of bias. It is acknowledged that a proportion of patients declined to have further tests but this group did not differ at baseline significantly from those followed up so it is unlikely that this was a significant source of confounding. In particular it should be noted that a similar proportion of those followed up (36%) and those not followed (37%) had fat free mass depletion at baseline. We think it is unlikely therefore that the findings of this study would have been skewed by an uneven pattern of drop out.

We chose to define exacerbations as episodes of worsening of disease sufficient to cause patients to seek medical assistance and receive a prescription for antibiotics. This definition can therefore to some extent be criticized as dependent on behaviour. On the other hand it has the merit of incorporating an element of 'clinical significance'. Other definitions and techniques such as diary cards have been used and this remains an area of controversy, but to date no consensus exists in the literature as to which is the 'gold standard'. It seems unlikely that a different definition would have caused any systematic difference in the results obtained. In addition it was possible in most patients to correlate their reports of exacerbations with the medical notes relating to clinic attendances in the intervening year to increase accuracy. As part of their routine clinical care at clinic visits during the year patients had been asked to recall exacerbations treated at home. Thus at the end of the year a 'contemporary' record of events was available to correlate with patients' recollection.

Bioelectrical impedance analysis has been shown to be highly repeatable on consecutive days in patients with COPD [30]. In healthy subjects, isometric quadriceps force had a 95% repeatability coefficient of 7.6 kg in a study of healthy controls with a mean strength of 93 kg [31]. Limited data are available about the repeatability of measures of quadriceps strength in this patient group. QMVC measured on two occasions within 2 weeks of each other in our lab in a group of 15 patients with COPD was 28.8(9.2) at baseline and 29.9(9.8) at 2 week follow up with a Bland Altman coefficient of repeatability (1.96 times the SD of the difference between the measurements) of 6.0 kg [32]. Given the gap between study visits it is unlikely that there would have been a significant learning effect to bias the results.

Activity levels might also be expected to impact on changes in strength and body composition but these data were not collected in this study.

### Significance of findings

To our knowledge this is the first prospective study looking at skeletal muscle impairment in patients with COPD over a significant period of follow up. Other studies have been short term [33,34], or where the effect of a therapeutic intervention such as growth hormone or anabolic steroids has been studied, the control group has also undergone pulmonary rehabilitation [35-37].

Exacerbations of COPD are known to be associated with negative nitrogen balance and elevated levels of cytokines [15] and an association between systemic inflammation and fat free mass depletion has previously been noted in clinically stable COPD [38]. However frequent exacerbations were not retained as an independent predictor of FFM decline. This may be because mechanistically it is in fact the prevailing 'stable state' that is more important than these acute episodes. Patients with a higher FRC are likely to have a greater work of breathing continually and to be more limited by breathlessness. Alternatively, since exacerbations tend to occur more frequently in more severe disease it may be that our study was not large enough to pick up a discrete exacerbation 'signal' among other co-varying markers of disease severity such as FEV_1_, SGRQ and in particular FRC.

Oral corticosteroids have been proposed as a significant cause of skeletal muscle impairment in COPD [12] although other studies have not found a correlation with strength [13,39-41] and short courses in stable patients do not appear to have any significant effect on muscle function [32]. Our study adds to the evidence that maintenance oral steroid treatment may be harmful in COPD with a significantly greater decline in FFM in this group. Maintenance therapy has been shown to attenuate the improvement in muscle bulk occurring with nutritional supplementation during pulmonary rehabilitation [42] and to increase the risk of death [43,44]. In the group as a whole, who mostly received only short burst treatment with corticosteroids, there was no association between steroid exposure and fat free mass or strength, either at baseline or during the follow up period, suggesting that the latter strategy is less harmful. It remains possible that maintenance corticosteroid treatment was a surrogate for a history of frequent exacerbations.

The benefits of smoking cessation on decline in lung function are well established [45]. Our data suggests an additional benefit with a significant increase in FFM occurring in the quitter group. Although weight gain following smoking cessation is commonly described we are not aware of any data showing an increase in FFM in COPD patients who quit. The mechanism for this benefit apart from increased appetite or exercise could be a reduction in the systemic inflammation that is present even in apparently healthy smokers [46].

Sniff nasal inspiratory pressure did not decline during the course of this study. The diaphragm in COPD experiences an increase in loading in contrast to the lower limb muscles where disuse is an important feature. Our findings are consistent with the view that systemic factors such as inflammation are relatively unimportant in the aetiology of muscle weakness or else that they have a synergistic effect with disuse which spares the inspiratory muscles but impacts on muscles of locomotion.

It should be noted that by the time patients were enrolled in this study they had already developed significant weakness with mean QMVC only 66 percent predicted. In addition more than a third of them had significant nutritional depletion. Given that baseline FFM was strongly correlated with quadriceps strength at baseline, these two factors are clearly linked even if change over the period of follow up appeared to be dependent on different factors.

A further question will be to investigate the interaction between COPD and other co morbidities that occur frequently in these patients and also impact on skeletal muscle such as heart failure and vascular disease.

### Conclusion

This study demonstrates a reduction in quadriceps strength over one year of follow up greater than would be anticipated in a healthy population. We do not know if there is early rapid loss of strength which then slows or if the decline is steady or if it is stepwise, perhaps in the context of exacerbations. The pattern of decline may well differ in different disease phenotypes. Our model explained only 46% of the decline in fat free mass over one year. Because our patients were recruited from hospital rather than primary care the population was inevitably weighted towards patients with more severe disease and it is clear that in order fully to understand the aetiology of muscle weakness and fat free mass depletion, future studies will need to enrol patients at an earlier point in the disease process and for longer periods of follow up. This should make it possible to understand better the role of factors such as systemic inflammation or hormonal depletion.

## Abbreviations

ADD average daily dose of prednisone

BMI body mass index

COPD chronic obstructive pulmonary disease

FEV_1 _forced expiratory volume in one second,

FFM fat free mass

FFMI fat free mass index

FRC functional residual capacity

PEmax maximum expiratory pressure,

QMVC quadriceps maximum voluntary contraction

SGRQ St George's respiratory questionnaire

SNiP sniff nasal pressure

TL_CO _carbon monoxide transfer factor

## Competing interests

The author(s) declare that they have no competing interests.

## Authors' contributions

NSH, MIP, JM and TTH conceived the study, NSH drafted the original manuscript. NSH, RCT, MJD, EBS took part in data collection. All authors have been involved in interpretation of the data and have seen and approved the final version of the manuscript.
